# Patient Involvement in the Development of Clinical Practice Guidelines – An Interview Study on the Experiences of Patients and Their Representatives in Germany

**DOI:** 10.1002/gin2.70081

**Published:** 2026-08-10

**Authors:** Clara Orduhan, Stefanie Pfisterer‐Heise, Käthe Goossen, Jessica Breuing, Dawid Pieper

**Affiliations:** ^1^ Institute for Health Services and Health System Research, Faculty of Health Sciences Brandenburg, Brandenburg Medical School (Theodor Fontane) Rüdersdorf Germany; ^2^ Center for Health Services Research, Brandenburg Medical School (Theodor Fontane) Rüdersdorf Germany; ^3^ Institute for Research in Operative Medicine (IFOM), Faculty of Health, School of Medicine Witten/Herdecke University Cologne Germany

**Keywords:** clinical practice guideline, communication, consultation, Germany, participation, patient involvement, patient representatives

## Abstract

**Background:**

Patient involvement (PI) in the development of clinical practice guidelines (CPGs) is recognized as a key aspect of trustworthy and high‐quality CPGs. However, difficulties exist and PI is not yet consistently implemented in the development of CPGs. Our study aimed at exploring the experiences and perspectives of patients and their representatives regarding their involvement in CPG development processes and to unravel existing challenges.

**Methods:**

Qualitative semi‐structured interviews were conducted with patient representatives who already participated in the development of a German evidence‐ and consensus‐based CPG. The interviews were conducted using a semi‐structured interview guide developed a priori. They took place via the online meeting tool WebEx and were digitally recorded. The interview data was analyzed using qualitative content analysis as described by Mayring using MAXQDA.

**Results:**

In total, we conducted 23 interviews from November 2024 to February 2025. Participation in CPG development processes as active and contributing member of the guideline development group was emphasized as the dominating involvement strategy. For a positive PI experience, participants emphasized several influencing factors such as preparatory training, participation of other patient representatives, and appreciation from other guideline group members. Moreover, several challenges were anticipated for the future, for instance the recruitment of patients as well as the need for a more inclusive and diverse involvement.

**Discussion:**

Our study found that overall participants were satisfied with the predominant involvement method of acting as participatory members in the guideline development group. Nevertheless, the findings also revealed certain unmet needs. Preparatory activities, such as targeted training sessions or meetings, were identified as essential and should become standard practice. To ensure equitable implementation, CPG developers should consider taking responsibility for preparing patient representatives to participate effectively, rather than relying on patient organizations for this.

## Summary

- Patient representatives highlighted active participation as full, contributing members of the guideline development group as the dominating involvement strategy.
- Preparatory training, participation of other patient representatives, and appreciation from other guideline group members were associated with a more positive participation experience.
- Participants emphasized the importance of inclusive and diverse patient involvement.

- **What this adds to what is known?**
  ∘ A novel finding of our study is the association between the gender of patient representatives and their experience with participation. Being female was described as a potential barrier to a positive participation experience, especially when being faced with a predominantly male guideline panel.
  ∘ Whereas previous interview studies reported that patient representatives often preferred patient‐exclusive reference groups over acting as participatory members in consensus conferences of the whole guideline development group, participants in our study expressed contrary preferences by emphasizing the high value of collective exchanges within the full guideline development group.
- **What is the implication and what should change now?**
  ∘ Further cross‐country and cross‐health‐system research is needed to clarify under which circumstances or contextual conditions, patient‐exclusive focus groups may be appropriately prioritized over active participation, and when they should not.
  ∘ Preparation in the form of training, consultation or the provision of preparatory materials should become standard for both patient representatives and other members of the guideline panel.
  ∘ Raising awareness of gender‐sensitive aspects, especially across guideline panelists who have a professional health background, should be integrated into preparatory materials and training.

## 1 Introduction

The Guidelines International Network (GIN) as well as the Appraisal of Guidelines Research and Evaluation II (AGREE II) recommend incorporating patients' values and preferences into the development of clinical practice guidelines (CPGs) [1, 2]. Internationally, involving those affected is recognized as a key aspect of trustworthy and high‐quality CPGs [1, 3]. It ensures more appropriately phrased recommendations as well as greater acceptance of these [1, 3]. This can lead to CPGs having a stronger impact on health decision‐making processes [4, 5, 6]. However, difficulties exist and patient involvement (PI) is not yet consistently implemented in the development of CPGs [6, 7, 8].

Studies found ambiguities regarding the ideal stage and method of PI [4, 9, 10, 11]. With regard to the extent and timing of involvement, there is debate as to whether involvement in the entire process has advantages over partial involvement [9, 12, 13, 14, 15]. Choosing the appropriate methodology for PI is even more complex, that is, do patients participate in the guideline development group or are their perspectives gathered by using qualitative and/or quantitative research methods [4]. To answer these questions, GIN has published a toolkit that deals with the involvement of patients and the public in CPGs [1]. According to this toolkit, there are three strategies of involvement: consultation, participation and communication (including implementation) [1]. Consultation aims at collecting perspectives from a broad range of patients, for instance by conducting surveys, interviews, or focus group discussions [1]. Participation describes the active involvement of patients as development partners, that is, as members of the guideline development group [1, 4]. This approach seems to be the most commonly used and preferred approach as patients and their representatives have the right to participate in discussions on the design of recommendations for action and other content and often also have a mandate to vote [1, 16, 17]. Communication comes into play at a later stage of CPG development, as it describes implementation and dissemination strategies, for example, providing content in lay language and/or patient versions of CPGs [1].

The experiences of patients and their representatives participating in the CPG development process have been investigated in recent years in only a few studies with heterogeneous results [4, 9, 18, 19]. A report on CPGs developed by the National Institute for Clinical Excellence (NICE), published in 2004, summarized that patient representatives were overall satisfied with their active participation in guideline development [18]. Findings from a German interview study, published in 2014 highlighted three main categories associated with a positive participatory experience: knowledge, appreciation, and adjusting to the role of being a patient representative [9]. In 2022, Finish group interviews found that patient representatives preferred patient‐exclusive focus groups over active participation in guideline development groups [4]. A recently published qualitative study from Australia highlighted the need for supporting patient representatives throughout the CPG development process [19]. However, issues identified in these studies seem to persist.

This is why our qualitative interview study aims to take a broader perspective and to understand:

1. What current practices of PI in CPG development are employed in Germany, that is, at what stage and with which method patients and their representatives such as family members, carers or advocates nominated by patient organizations (in the following summarized as: patient representatives) were involved in the CPG development process.
2. What roles and responsibilities patient representatives had during the process, specifically compared to other members of the guideline development group.
3. How patient representatives perceived the CPG development process, specifically with respect to their involvement.
4. What challenges patient representatives see for the future of PI in CPG development.

## 2 Methods

Our study used qualitative semi‐structured interviews to explore the experiences and perspectives of patient representatives regarding their involvement in CPG development processes. We followed the recommendations of the Consolidated Criteria for Reporting Qualitative Research (COREQ) checklist to report this study (Supporting Information S1). The characteristics of the research team are described in Supporting Information S2. A study protocol was published a priori on OSF [20].

### 2.1 Participants and Recruitment

We purposively recruited participants with the criteria of (a) being a patient representative who (b) had already participated in the development of at least one German CPG, based on both evidence and consensus (S3‐level CPG), which represents the highest level within the German CPG classification system [21]. S3‐level CPGs combine evidence and consensus, that is, systematic literature review and evidence synthesis, as well as a structured consensus process involving members of a representative guideline development group [21]. To achieve this, we initially reached out via email to patient representatives (*n* = 6) known to the research team. Subsequently, we contacted German patient organizations (*n* = 12) that had previously nominated patient representatives for at least two valid S3‐level CPGs. To identify suitable organizations, we drew on the results of a previously conducted meta‐research study on PI in CPG development in Germany [6]. Via email, we sent out information material on our project and inquired whether there were patient representatives within these organizations who met the eligibility criteria and were interested in participating in an interview. Additionally, after each conducted interview, we asked the interviewee for potential study participants. We did not collect reasons for non‐participation. We recruited until the interviewer received no new information during the data collection process and meaning saturation was reached [22]. In addition to the informed consent form, all participants completed a short questionnaire beforehand, which included questions on sociodemographic data as well as on their individual participation experience in CPG development (number of participations, topic of respective CPGs, prior experience in scientific or guideline related projects).

### 2.2 Interview Guide

We developed a semi‐structured interview guide (Supporting Information S3) based on this study's objectives as well as on the content of the GIN Public toolkit and other relevant literature [1, 4, 9, 18]. An initial questionnaire was drafted by one researcher (CO), then revised and refined based on the research team's feedback (SPH, KG, JB, DP). We piloted this refined version in a participatory process with two patient representatives who already had been recruited and agreed to participate in this study. First, we discussed the interview guide with one patient representative, explained our objectives, and asked for feedback. The feedback was implemented and incorporated. This patient representative did not participate in the interviews. In a second step, we conducted a pilot interview with another patient representative. After the interview, we again asked for feedback. As no further adjustments to the interview guide were necessary, this pilot interview was included in the data analysis.

### 2.3 Data Collection

The interviews were conducted in German by one interviewer (CO) using the semi‐structured interview guide. They took place via the online meeting tool WebEx and were digitally recorded. Before recording was started, the interviewer introduced herself, briefly summarized the study's objectives and reminded the participants of their right to withdraw at any point. The interview records were anonymized and transcribed verbatim using the software noScribe (version 0.5). Transcripts were not returned to participants for review or correction. One researcher (CO) conducted a quality check and proofread of the transcripts to ensure accuracy and correctness. Additionally, field notes were made during or right after the interviews to memorize important information. No repeat interviews were carried out and no participants dropped out.

### 2.4 Data Analysis

Two researchers (CO, SPH) coded and analyzed data using qualitative content analysis as described by Mayring [23] with MAXQDA 2022 analysis software. A category system was developed by using a deductive‐inductive approach and then discussed within the research team. We created a coding tree including the names of each category, sub‐category and sub‐sub‐category as well as a brief definition, an anchor example, and if necessary, a coding rule (Supporting Information S4). The finalization of the category system and the coding process was conducted iteratively and included the following steps: (a) familiarization with the interview transcripts and development of an initial coding tree (CO), (b) discussion of the coding tree and adaptation of the initial version (CO, SPH), (c) independent coding of a randomly selected sample of three interviews by two researchers followed by a discussion to refine and finalize the category system; comparing results during discussion (CO, SPH), (d) independent coding of a randomly selected sample of two other interviews by two researchers using the final version of the category system (CO, SPH), (e) all subsequent coding was performed by one researcher (CO) as the independent coding of the last two interviews conducted by two reviewers (CO, SPH) showed an appropriate interrater reliability across all coding categories (kappa > 0.7). Another researcher (DP) provided support if needed. To display quotes within the results section, we translated relevant parts of the transcripts with the software deepl.com. Translated quotes were reviewed by all authors to ensure their accuracy. Interview participants were not asked to provide any feedback on the final coding or findings.

## 3 Results

In total, we conducted 23 interviews from November 2024 to February 2025. The duration of the interviews varied between 27 min and 1 h 15 min, with a mean of 45 min. Participants came from 15 different patient organizations. They were heterogeneous on whether they had lived experience with the condition discussed in the respective CPG, but also in terms of gender, number of CPG development participations, and experience in scientific or guideline related topics before their first involvement in guideline development. Most of the participants identified themselves as patient representatives or patients, and only one as family member of a patient. The participants mainly had a university degree or higher. Their mean age was 63 years (range: 48–84). Characteristics of all participants are reported in Table 1.

**Table 1:** Characteristics of interview participants.

	Number (*N* = 23)
Role*	
Patient representative	22
Patient	2
Family member of a patient	1
Lived experiences with condition discussed in CPG(s)	
Yes	9
No	6
Varies (in case of multiple participations)	7
n.r.	1
Gender	
Female	14
Male	9
Age [years]	
< 65	12
≥ 65	11
Level of education	
Intermediate secondary school	4
University degree	16
PhD	3
Number of CPG development participations	
≤ 2	9
> 2	14
Experience with CPGs/scientific projects before first CPG development	
Yes	14
No	9

* Numbers do not sum up to 23 as multiple selection was possible.

The results are presented according to this study's research questions: current practice of PI including patient representatives' roles and responsibilities, patient representatives' perceptions and experiences, and future challenges.

### 3.1 Current Practice, Roles and Responsibilities

Current practice regarding PI in CPG development processes varies (see Figure 1). The only consultatory involvement strategy employed was patient‐exclusive focus groups that took place prior to the commencement of the guideline development group's work. In the experience shared by one patient representative, they got the chance to *“meet other patient representatives and hear what kind of knowledge or experience they have”* [I1], which then led to fruitful discussions, and encouraged patient representatives to participate in the subsequent discussions with other guideline group members, that is, in exercising their active participation.

**Figure 1:**
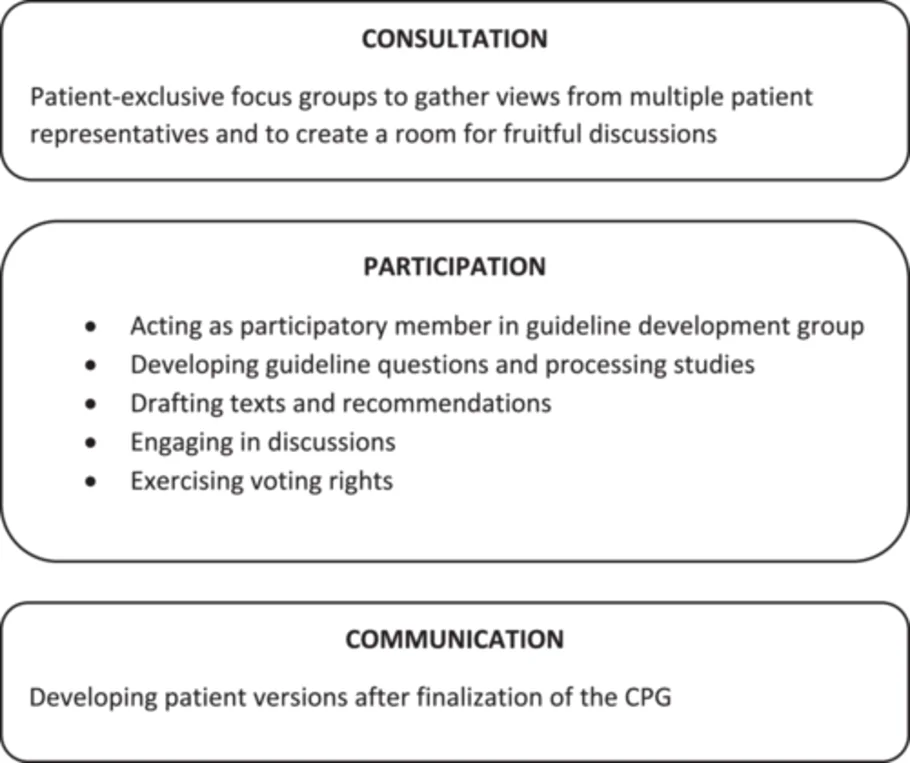
Current practice of PI in the development of CPGs in Germany.

Participation was the dominant involvement strategy. The role of patient representatives was to act as contributing members of the guideline development group. This involved, in particular, reading and drafting texts and recommendations, providing comments on content, and engaging in discussions. Additionally, patient representatives exercised voting rights in the final ballot and participated in the consensus conferences. Even though participants in this study mostly saw themselves as guideline group members with equal status, differences in expertise, knowledge, and educational levels between medical doctors and patient representatives were also frequently mentioned.

You have just as much of a voice as the others, only that yours is a layperson's voice, while the others are expert voices.

[I2]

Some participants mentioned that their involvement even went further and described their role as *“partners cooperating on an equal footing”* [I10], who were involved in every single step of the development process, for example, in the development of CPG questions and the processing of studies. Others, by contrast, were hardly actively involved and therefore, could not contribute as much. They identified themselves as more of a “*token figure or a fig leaf for patient legitimacy”* [I12].

Communication as involvement strategy was barely mentioned. Some participants highlighted the benefits of being involved in the development of patient versions of CPGs and their usefulness.

### 3.2 Perceptions of Patient Representatives

Overall, participants were satisfied with acting as participatory members in the guideline development group. Consultatory methods such as individual interviews would undermine *“the value of a consensus conference [which] lies precisely in this collective exchange with one another”* [I15]. Moreover, participants agreed on the positive impact that PI has on the CPG itself as well as on the development process.

[…] where we simply contributed clearer phrasing. And we're still a bit proud of, for example, the wording on patient organizations providing support in decision‐making […]. That was originally us.

[I6]

Additionally, study participants emphasized several influencing factors for a positive PI experience (see Table 2). Earlier participation in CPG development (i.e., previous experience), preparatory training/meetings, participation of other patient representatives, and respectful behavior as well as appreciation from other guideline group members were emphasized as facilitators for a positive participatory experience. Additionally, internal factors, for example, self‐confidence and the ability to argue, as well as external factors such as high‐quality moderations and the backing of a relatively large, structured, and well‐organized patient organization were identified as facilitators.

**Table 2:** Factors that influence patient representatives' CPG development experience.

Influencing factor		Quotes
Earlier guideline participations	Facilitator	*“So, when you first start out, it can all feel a bit overwhelming and you might think, ‘What am I actually doing here?’, because it is all very specialized and very technical.”* ‐ I9
Preparatory training/meeting	Facilitator	*“We also had separate meetings at the start to clarify, as you said, what our role would be and what exactly we were expected to do. Over the course of the process—which I really appreciated—we repeatedly received information prepared specifically for the patient representatives. […] Just two weeks ago, when the allocation of studies was discussed, we received a video that explained everything in detail: how the process works on the portal, what steps we needed to take, and what our tasks were. […] there was a genuine effort to include us, with special training sessions and meetings organized just for us.”* ‐ I10
Participation of other patient representatives	Facilitator	*“So, for me, getting started was relatively easy because I knew a patient representative who was already very experienced, and she more or less guided me into it.”* ‐ I4
Respectful behavior from other guideline group members	Facilitator	*“There is no condescension there; instead, everything is explained to you with great effort, and that works well. So you do not need to be afraid to ask questions, no matter where you are there.”* – I2
Appreciation from other guideline group members	Facilitator	*“He [chair of the guideline group] spoke to both of us and said, ‘You really need to remember this: you are extremely important here and we need your experience.’ That made a deep impression on me.”* ‐ I19
Self‐confidence and the ability to argue	Facilitator	*“So, I find it difficult to contribute to the discussion in the guideline process, because healthcare professionals are predominantly present. I also tend to assume that they simply know better than I do […].”* ‐ I22
Quality of moderation	Facilitator	*“Every meeting is […] moderated, but some people are not very good at it. The necessary skills include giving space […] to all perspectives, continuously translating and summarizing, and also […] making sure that it is clear whether what each side wants has actually been understood.”* ‐ I22
Backing of patient organization	Facilitator	*“What we [respective patient organization] do today, when we appoint someone, is that we make a point of spending time with them beforehand—there's no question that we provide them with training in advance. [We have] a working group on patient involvement in healthcare, […] where we introduce these things to people.”* ‐ I3
Use of technical terms	Barrier	*“This scientific language that is mainly used, with all the many foreign terms, really causes you trouble at the beginning.”* – I2
Gender	Barrier	*“At the beginning, it was really like that — I was sometimes the only woman sitting among suit‐wearing men, said beep very carefully.”* * ‐ I1
Online meetings	Mixed influencing factor	*“Attending in person is, of course, much more demanding, especially for those affected and for severely ill individuals who may no longer be physically able to travel. In such cases, an online format is considerably easier to implement.”* ‐ I15 *“And especially when you're in a Zoom meeting with ten or twenty people, it's easy to forget — oh right, there's someone else there too.”* ‐ I7

* German idiom which, in terms of meaning, expresses that one hardly dares to say anything.

Study participants also mentioned barriers for a positive participatory experience. The first challenge identified was the use of technical terms and the difficulty of understanding them. Both in relevant documents and studies to be read, as well as during group discussions, scientific and medical language was frequently used, which posed a barrier to understanding for patient representatives without medical background. As a result, those participants often struggled to follow discussions, let alone contribute actively to them. Moreover, patient representatives' gender was associated with their participation experience. For instance, female participants were in some cases confronted with predominantly male committees, which according to participants may have adversely affected the extent of their engagement. In this case, gender was described as a possible barrier for a positive participation experience.

The increasing predominance of online meetings within the CPG development process was assessed ambivalently. On the one hand, this format enables a low‐threshold and inclusive participation, particularly for patient representatives. On the other hand, in‐person meetings foster interpersonal communication, reduce the likelihood of patient representatives becoming overlooked within larger groups, and thereby facilitate their active engagement in discussions.

### 3.3 Future Challenges

In addition to recounting their individual experiences with CPG development processes, participants also identified several challenges they anticipated with respect to the future implementation of PI in CPG development (see Figure 2). The responsibility for resolving or addressing those challenges was mainly attributed to the organizers and chairs of the respective guideline development groups and can be classified into four categories. The first challenge identified concerned the recruitment of patient representatives. This issue involved both motivating a sufficient number of patient representatives to participate and ensuring that those recruited comprehended their role as representatives of a broader patient group and refrained from focusing solely on their individual experiences.

Finding people who are willing to contribute to guideline development. But, as mentioned, this doesn't happen on its own. There's no one who simply raises their hand. Guideline developers also need to take an active role.

– I5

A patient representative who speaks only about their own situation is not a true patient representative […].

– I6

**Figure 2:**
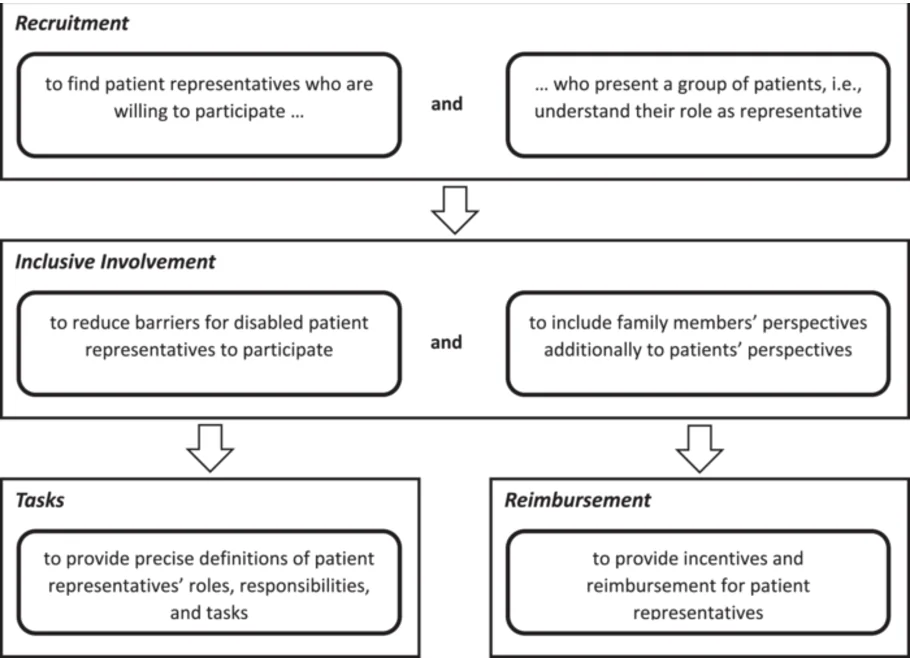
Identified challenges and tasks for guideline groups, guideline chairs and organizers of PI in CPG development.

Second, promoting more inclusive involvement was highlighted. In this context, it was emphasized that individuals with substantial impairments (e.g., speech difficulties resulting from aphasia) should also be actively involved. Moreover, there was a recurrent call to incorporate the perspective of (caring) family members in the development process alongside the patient perspective.

They [individuals with aphasia] lack the words to express what they would like to communicate. However, to me, that does not mean this issue should simply be dismissed. From an ethical and moral standpoint – or however one may put it – such exclusion is entirely unacceptable.

– I13

I think it's important to include not only the patient‐related outcomes but also the significant‐others‐related outcomes – and to give them equal weight.

– I13

The third challenge identified concerned the necessity of establishing clear and precise definitions of patient representatives' roles, responsibilities, and tasks. This can also be linked to the challenge of finding qualified patient representatives who understand and embrace their role as representatives.

But what is our role? And defining this role, I believe, is probably the most difficult part – for everyone involved.

– I7

Finally, financial reimbursement and incentives were emphasized. This challenge is gaining particular relevance, as it is closely associated with the recurring difficulty of recruiting patient representatives.

Where are they [patient representatives] supposed to come from if they do not even receive a small financial compensation […]? I mean, who still does that nowadays? Who goes to work and then volunteers an additional 5 or 6 h per day? We won't find people willing to do that anymore.

– I20

## 4 Discussion

Our study found that the majority of patient representatives perceived themselves as equal participatory member within the guideline development group, executing relevant tasks such as phrasing recommendations and texts, participating in discussions, and exercising voting rights. Consultatory engagement strategies such as patient‐exclusive focus groups were uncommon, but also not explicitly wished for by the study participants. Being involved in the development of patient versions of CPGs and other communication strategies were highlighted as important – but only in addition to participating in CPG development itself, not in its place. In 2022 and 2026, interview studies with Finnish and Australian patient representatives found that patient‐exclusive reference groups were often preferred over acting as participatory member in consensus conferences of the whole CPG group [4, 19]. Our study's participants mentioned contrary preferences by emphasizing the high value of collective exchanges within the CPG development group. This rather positive perception of active participation may partly reflect familiarity with this format as most of our study participants did not experience consultatory involvement strategies. These findings align with recent literature on the most common involvement strategies [24, 25]. Further cross‐country and cross‐health‐system research is needed to determine the origins of these differing preferences and to explore when, that is, under which circumstances or contextual conditions, patient‐exclusive focus groups may be appropriately prioritized over active participation, and when they should not.

We also found several factors influencing the individual patient representative experience regarding involvement – both positively and negatively. These factors partly align with findings from previous studies on barriers and facilitators for the development of patient versions of CPGs, and also on concepts of interest‐holder engagement in CPG development [19, 26, 27, 28]. For instance, lack of consultation and lack of specific training were highlighted as barriers, as well as the need to increase self‐confidence in patient representatives in order to reduce power imbalances [19, 26, 27, 28]. One novel finding of our study was the association between the gender of patient representatives and their experience with participation. Being female was described as a potential barrier to a positive participation experience, especially when being faced with a predominantly male guideline panel [29]. This is particularly problematic in areas where female patient representatives are more likely to participate, such as healthcare research [29, 30, 31].

Furthermore, it is noteworthy that although our sample of participants was heterogeneous, individuals with a lower educational level were markedly underrepresented compared to those with a university degree or even a PhD. Moreover, all participants’ first language was German. This observation aligns with recent empirical findings indicating that individuals with lower educational attainment and socioeconomic status exhibit substantially lower levels of interest in participating in scientific research projects [32]. Even though no pattern emerged from our findings indicating that individuals with lower educational attainment reported more negative experiences, overall, a more diverse sample could potentially have led to less positive experiences. However, the distribution of our study's participants may also reflect the actual and predominant characteristics of patient representatives participating in CPG development in Germany in general [25, 29, 31]. Further research should be conducted on how to facilitate involvement and engagement of people with fewer opportunities, such as disabled persons and those with lower socioeconomic status.

To date, the exploration of patient representatives' perspectives in the development of CPGs remains scarce [4, 9, 18, 19]. Problem‐centered interviews with German patient representatives were already conducted in 2014 [9]. However, they mainly focused on the perception of patient representatives' own roles in German CPG development processes as well as on the communication flow within the CPG group [9]. In contrast, our study took a broader perspective on the current practice of PI, on experiences regarding the CPG development process, and on challenges regarding the future of PI in CPG development, all reported from patient representatives. It is noteworthy that already the study, published in 2014, explored the need for explicit descriptions and definitions of patient representatives' roles in the process as well as the necessity of training sessions [9]. More than a decade later, our study as well as recent research from Australia [19] found that these needs still exist. Even though guidance for PI in CPGs is available, internationally less than 40% of guideline organizations provide CPG training sessions for interest‐holders in general and even less for patient representatives in particular [24]. In Germany, a meta‐research study from 2025 found solely one CPG that reported to have conducted methodology training, however not for patient representatives in particular but for the whole guideline group [6].

Our findings have several implications for practice and research. Preparation in the form of training, consultation or the provision of preparatory materials should become standard for both patient representatives and other members of the guideline panel. Given that these findings have been known for over a decade but are still not implemented sufficiently into practice, future research needs to focus on determining and developing the ideal format and structural design of training materials, that is, materials that are well‐suited to the needs of the respective interest groups, resource‐efficient, and easily accessible and understandable. Raising awareness of gender‐sensitive aspects, especially across guideline panelists who have a professional health background, should be part of these activities and materials.

### 4.1 Limitations

This study is subject to certain limitations. First, due to regional variations and differences in healthcare systems, the transferability of the results to other countries and healthcare contexts is questionable. Nevertheless, our findings provide a better understanding of patient representatives' perspectives regarding PI in CPG development and contribute to potential improvement approaches, some aspects of which can certainly be applied to other contexts. Second, the unexplained discrepancies compared to the findings of the Finish and the Australian interview studies [4, 19] should be noted, as their findings revealed different preferences of patient representatives regarding their involvement method. The question arises whether patient‐exclusive focus groups or other alternative involvement methods should be made more widely known, not only among patients, but also among guideline development groups and their organizers and coordinators. Dissemination strategies for this purpose could be incorporated internationally through the GIN Public Toolkit [1] and, additionally in Germany, through the Manual of the Association of Scientific Medical Societies (Arbeitsgemeinschaft der Wissenschaftlichen Medizinischen Fachgesellschaften) [33]. Third, the sample included relatively few participants with lower educational background, most participants were of a relatively high age, and they were all native German speakers, potentially reflecting the tendency of patient representatives in CPG development to possess higher education levels. Moreover, we did not ask for sexual orientation and therefore cannot comment on the diversity of the sample in this regard. All these aspects may have led to selection bias. Greater heterogeneity in education, age, and diversity in terms of sexual orientation and cultural background would have been preferable. Fourth, to minimize barriers to participation in the interviews, we did not specify a time limit for when the participant's most recent involvement in developing a CPG should have taken place. However, this may have led to recall bias. Fifth, we recruited most participants via patient organizations, whereby we were dependent on which individuals were contacted by the organizations themselves. This may have led to bias regarding the frequently mentioned facilitating factor ‘backing from patient organizations.’

## 5 Conclusion

In summary, the majority of patient representatives served as active participatory members of the CPG development group and expressed satisfaction with this method of involvement. Nevertheless, the findings revealed certain unmet needs. Preparatory activities, such as targeted training sessions or preparatory meetings, were identified as essential and should be implemented, evaluated, and tested in different settings. To ensure equitable implementation, CPG developers should consider taking responsibility for preparing patient representatives to participate effectively, rather than relying on patient organizations for this. Furthermore, within the CPG group itself, efforts need to be made to increase awareness and competences in areas such as inclusive involvement strategies and moderation skills.

## Author Contributions

**Clara Orduhan:** conceptualization, methodology, investigation, formal analysis, visualization, project administration, writing – original draft, writing – review and editing. **Stefanie Pfisterer‐Heise:** conceptualization, methodology, formal analysis, writing – review and editing. **Käthe Goossen:** methodology, writing – review and editing. **Jessica Breuing:** methodology, writing – review and editing. **Dawid Pieper:** conceptualization, methodology, resources, writing – review and editing, project administration, supervision.

## Funding

The authors have nothing to report.

## Ethics Statement

The study received an ethics waiver by Brandenburg Medical School, Germany (waiver No. 251092024‐ANF). Participants were informed about the study's objectives, the duration of the interview (30–60 min), and data protection. Participants who agreed to take part signed a consent form and sent it back to the interviewer before the interview took place. No incentives were provided for participation.

## Conflicts of Interest

C. Orduhan, S. Pfisterer‐Heise, J. Breuing declare no conflicts of interest. K. Goossen and D. Pieper are members of GIN Public.

## Supporting information


Supporting File 1

Supporting File 2

Supporting File 3

Supporting File 4

## Data Availability

The data that support the findings of this study are available on request from the corresponding author. The data are not publicly available due to privacy or ethical restrictions.
