# Supplementary material for: Patient Involvement in the Development of Clinical Practice Guidelines – An Interview Study on the Experiences of Patients and Their Representatives in Germany
Source: Clin Public Health Guidel. 2026 Aug 10;3(4):e70081. doi: 10.1002/gin2.70081 (PMC13618489; doi:10.1002/gin2.70081)
Supplement: Supplementary file 2 [file GIN2-3-e70081-s001.docx]

**Supplementary Material 3 – Interview guide**

| **General outline** | **Specifying questions** | **Conversation and sustaining questions** |
| --- | --- | --- |
| **Introduction** | | |
| Introduction Project objectives Skip questions/ terminate interview Any questions? | My name is Clara Orduhan, and I am a research associate at the Institute of Health Services and Health System Research (IVGF) at Brandenburg Medical School. We are currently conducting the study “Patient Involvement in the Development of Clinical Practice Guidelines.”  The aim of this study is to examine the experiences you have had while being involved participating in the development of a clinical practice guideline, to explore how the guideline development process could be improved from your perspective, and to identify potential challenges. For this purpose, we would like to speak with you about your experiences.  If at any point during our conversation you wish to end the interview, you may do so freely. You can also skip any question you prefer not to answer, without any disadvantages to you.  Do you have any questions before we begin? |  |
| **RECORDING START** | | |
| **Block 1: Experiences with guideline development. Key question: Please describe your personal experiences related to developing or updating clinical practice guidelines.** | | |
| Understanding of the guideline development process  Memories and experiences | - What comes to your mind when you think back about being involved? - How did you feel during the process? - When you think back about being involved what was a particularly positive experience? - What was a particularly negative experience? - If you were involved multiple times: How did you experience the processes or your involvement in the different guideline developments differently? | Could you tell me more about that?  Could you please give an example?  What exactly do you mean by that?  Could you describe … in a bit more detail?  Did … play a role? |

| **Block 2: Patient involvement (methods of involvement, roles, and responsibilities). Key question: At which stages of the overall guideline development process were you involved?** | | |
| --- | --- | --- |
| Type of involvement / specific tasks  Method of involvement (equal position in the guideline group, separate discussion round only for patients, advisory role, etc.)  Timing | - In what ways were you involved? - At what stage in the development of the guideline were you involved? At what other stages? - What did your involvement look like in reality? - Were you the only patient representative involved in the development, or were there several? To what extent were you able to request/ask for the involvement of additional patient representatives? | Could you tell me more about that?  Could you please give an example?  What exactly do you mean by that?  Could you describe … in a bit more detail? |
| **Key question: What tasks and responsibilities did you have in the guideline development process?** | | |
| Equal position in the guideline group, discussion round only for patients, advisory role, etc.  Clear communication about role  Consideration of patient perspective in the guideline | - Were tasks and responsibilities defined in advance?   If yes:   - How were the tasks defined? - Who was involved in defining them? - Were these definitions followed throughout the process? If not, what changed? If yes, in what way?   If no:   - To what extent was it clear to you what your role was? - In what ways did your role differ from those of the other members of the guideline group, and in what ways was it similar? - How was the patient perspective considered in the final version of the guideline? |  |
| **Key question: How did the other members of the guideline development process behave toward you?** | | |
| Opportunities for involvement  Support from the chair through good moderation  Acknowledgement of patient perspective  Awareness of other members in the guideline group | - To what extent did you have the opportunity to express your opinion and perspective? What role did the chair of the guideline group or working group play in this? - To what extent were your comments and perspectives heard and acknowledged? - To what extent do you think the other members of the guideline group were aware of what your role was? |  |
| **Block 3: Support Key question: To what extent did you receive any support before starting your involvement in the guideline development process(es)?** | | |
| Prior training  Wishes for prior support | - In the questionnaire we sent you beforehand, you indicated that you had (no) prior experience with guidelines or other scientific projects before being involved in guideline development. Did you receive any preparatory training at any point? - If yes:   - Who conducted the training?   - At what point did it take place?   - To what extent did it make you feel better prepared? - If no:   - What kind of support would you have wished for in advance? | Could you tell me more about that?  Could you please give an example?  What exactly do you mean by that?  Could you describe … in a bit more detail?  Did … play a role?  Do you mean … (summarized)? |
| **Key question: To what extent did you receive any support during the guideline development process?** | | |
| Information, people, procedures  Wishes for support | - Which people or processes/procedures helped you during guideline development? - If yes:   - From whom did you receive this support?   - To what extent did it make you feel better informed? - If no:   - What kind of support would you have wished for during guideline development? |  |
| **Key question: To what extent did you feel acknowledged for your contribution?** | | |
| Compensation (monetary or other)  Appreciation, public recognition of the guideline group  Wishes for the future | - In what ways did you perceive this acknowledgement? - To what extent do you consider financial compensation to be a form of acknowledgement? - What type of compensation would you wish for when being involved in the future? - If you did not feel acknowledged: How did that make you feel? What kind of compensation would you have wished for? |  |

| **Block 4: Challenges and the future Key question: Which aspects of the guideline development process did you find good or bad in terms of your involvement?** | | |
| --- | --- | --- |
| Process improvements  Alternative methods (literature review, survey, PICO design, literature suggestions [patient preferences] and evaluation, etc.)  Behavior of members of the guideline group  Positive experiences  “After the guideline is before the guideline”  Patient feedback | - If you could choose freely, in what way would you have preferred to be involved in the guideline development process? - What other forms of involvement, besides patient participation in the guideline group or individual working groups, would have been conceivable for you? - What would be necessary for such an alternative form of involvement? How would the other members of the guideline group need to behave? - What did you already like very much about the process? What made you feel comfortable? What advice would you give other patient representatives about involvement in guideline development? - To what extent was there further contact with other members beyond the guideline development process, particularly for planning future updates or similar activities? | Could you tell me more about that?  Could you please give an example?  What exactly do you mean by that?  Could you describe … in a bit more detail?  Did … play a role?  Do you mean … (summarized)? |
| **Block 5: Conclusion** | **Would you like to add anything to our interview? Are there any aspects that are important to you but have not yet been discussed?** |  |
| **RECORDING STOP** | | |
| Other contacts  Further contact in case of questions | - Do you know other patient representatives who have already been involved in guideline development and whom I could contact? - May I contact you again if any questions arise? |  |
