# Supplementary material for: Patient Involvement in the Development of Clinical Practice Guidelines – An Interview Study on the Experiences of Patients and Their Representatives in Germany
Source: Clin Public Health Guidel. 2026 Aug 10;3(4):e70081. doi: 10.1002/gin2.70081 (PMC13618489; doi:10.1002/gin2.70081)
Supplement: Supplementary file 3 [file GIN2-3-e70081-s002.docx]

**Supplementary Material 4 – Coding tree**

| **Category label** | **Sub category** | **Sub-sub category** | **Definition** | **Anchor examples** | **Coding rules** |
| --- | --- | --- | --- | --- | --- |
| Experience with guideline development | Perceived Impact of patient involvement on guideline |  | what kind of impact does patient involvement have (e.g. does the wording of a recommendation changed because of PI) | "And yes, I had already been able to influence the discussion in a certain direction on certain points that were in the interests of the patients, not just once, but several times, so to speak, and this was then actually reflected in the outcome, yes." |  |
|  | Perceived Impact of patient involvement on patient |  | what kind of impact does patient involvement have on the patients themselves (e.g. gain of knowledge) | "That's also what my colleague says, one of us who is also actively involved in other areas, who has been doing it even longer than me, much longer, and she always comes out with new knowledge and it's a pleasure for her to be there again and again." |  |
|  | factors that influence guideline development experience | Backing of patient organization | Factors that influence different experiences with CPG development processes, such as level of education, gender issues, or experience in other CPG development groups | "We have written an SOP for the guidelines for us, women's self-help organization, almost 15 pages of PowerPoint. And it says something like this. And really quite simply in German." | code only the sub-sub category |
|  |  | several participations/ experience |  | "In the beginning, it was really the case that I sat there, sometimes even as the only woman among men wearing black, and said beep very carefully. And today I am recognized as a member of the guideline group." |  |
|  |  | level of education/ability to argue |  | "This scientific language, which is mainly spoken here, the many foreign words, can be difficult at the beginning. And it's always a good idea to have your cell phone next to you and then search inconspicuously to translate the words. You can also ask directly, but you don't want to disturb the flow of information every time."  "And not to be put off, but to take objective countermeasures, that is also a process that I had to learn." |  |
|  |  | Size of guideline development group |  | "And as I said, then it goes into the sub-working groups, so it's not so big, it's clearer and it's easier to have a say in things." |  |
|  |  | gender |  | "In the beginning, it was really the case that I sat there, sometimes even as the only woman among men wearing black, and said beep very carefully. And today I am recognized as a member of the guideline group." |  |
|  |  | online meetings |  | "That has improved, but I think it's also down to the medium, i.e. video conferencing and adequate moderation. These video conferences take away the fact that this game simply doesn't take place on the sidelines." |  |
|  |  | ability to gain acceptance |  | "And that means that you have to get used to being accepted by the other camp, to which you don't quite belong mentally or professionally, and you have to work at it and you need a thick skin." |  |
|  |  | quality of moderation |  | "That has improved, but I think it's also down to the medium, i.e. video conferencing and adequate moderation." |  |
|  |  | participation of other patients |  | "And, uh, that's why it was relatively easy for me to get started because I knew a patient representative who was, uh, very experienced and then I was more or less, um, uh, led to it." |  |
| Advices for patients |  |  | advices for other patients about participating in guideline development and to support good patient involvement | "Prepare yourself well. [...] Have the courage to go there and be authentic."  "It's important that you have the courage to say something and that you really do that as a patient representative when all these texts come later, because you have to read through them again, so that you can have your say."  "In my experience, it's always good to start by saying a few personal words to the chair of the meeting. [...] They can remember my face and then they usually write down my name. So I've noticed that this is incredibly helpful." |  |
| Patient involvement | Stage of involvement ('when') |  | At what point in the CPG development were patients involved. | "The guideline consists of umpteen pages and we have a chapter."  "Then the preliminary vote takes half an hour, three quarters of an hour and then, of course, the guideline meetings, which are longer. They last 3-6 hours. And then there are 2-3-4, depending on how much the experts agree." | code only the sub-category |
|  | Method of involvement ('how') | Participatory member of the guideline development group | Method of involvement (e.g. Focus Groups, interview, participatory member of the CPG development group, ..) | "And as I said, then it goes into the sub-working groups, so it's not so big, it's clearer and it's easier to have a say in things." | code only the sub-sub category |
|  |  | Focus Group |  | "So we have a focus group in a guideline with Prof. Dr. H. And it's all about complementary medicine. And I think that's really great, because it allows me to get to know other self-help representatives and find out what their level of knowledge is. We also exchange ideas with each other." |  |
|  | Rights |  | rights of patients in the process of CPG development (e.g. voting rights) | "When I go into a guideline with my deputy, I say hello, I have my deputy with me. It's clear to us that we only have one vote and then it's immediately yes and then who would you like to vote for, Ms. S. or Ms. H. So today, that's no longer an issue at all. We have very equal rights." | code only the sub-category |
|  | Tasks/Responsibilities of patients |  | tasks and responsibilities of patients in the process of CPG development (e.g. own preparation) | "I read through the documents, I read through the chapter that deals directly with patient education."  "When all these texts come later, because you have to read through them again, so that you can have your say." |  |
|  | Tasks/Responsibilities of patient organizations |  | tasks and responsibilities of patient organizations in the process of CPG development | "One is to find people who enjoy it, who are qualified for it. And qualification, I think, also means being able to open your mouth at this point." |  |
| Behaviour/Attitude of the guideline panel towards patient | chair of the guideline panel |  | behaviour of the chair of the guideline group towards patient; role of chair to support PI | "When I come to a group that doesn't know me yet, they say, usually from the head of the commission, yes, we have a new patient representative today, Ms. H. is here, I would like to welcome her. Can you say a few more words?" | code only the sub-category |
|  | other (medical) members of the guideline panel |  | behaviour of other (medical) members of the guideline group towards patient; role of other (medical) members to support PI | "So, the most important thing I saw and experienced there was just being accepted into this large group of doctors, which was completely equal compared to all the things I had participated in so far, who were also prepared to explain things to me that I didn't know yet."  "There was that too. But the speakers, the individual contributions to the discussion, always emphasized the importance of the respective clinic where they came from. Always along the lines of, we're the best anyway. That took some getting used to for me at the beginning." |  |
|  | other patients of the guideline panel |  | behaviour of other patients of the guideline group towards patient; role of other patients to support PI (e.g. were other patients involved) | "So I'm the only one in the guidelines I mentioned. There's no other patient representative in there apart from me, apart from my deputy." | code only the sub-category; code also if there is **information about no other patients being part** of the guideline group |
| Experienced support for patient/patient organizations | training |  | received any preparatory training (yes/no/how) | "Unfortunately not at all [a training]. We have already asked the AWMF from the women's self-help organization, so I did it myself, because I had very good contacts through the many guidelines and somehow they didn't get around to doing it. " | code only the sub-category;   code both, if experienced or not experienced;  make sure to not double with "patients' wishes" categories |
|  | financial compensation |  | received any financial compensation (yes/no/details) | "It used to be the case that the professional associations covered both travel costs and accommodation costs. In other words, they didn't cover the time spent, but they did cover travel and accommodation costs. Today, they hardly have any money to cover accommodation costs. They cover travel costs, but no more accommodation costs." |  |
| Patients' wishes for future guideline development | alternative participation methods |  | other types of participation in CPG development (besides the classic ones) | "If they notice that the patient's perspective has been very thin or has not been voiced loudly, they might offer to hold a separate meeting where only the moderator is with that side." | **Code only if something like: “I wish...”, “I would like to have...”, “That would help me...” was explicitly said.**  code only the sub-category;   code both, if wished for or exlicitely not wished for; |
|  | wish for immaterial support |  | wished immaterial (i.e. non-financial) support in advance or during the CPG development | "And I was involved in so many working groups in the last round, where other patient representatives were actually also named, but they didn't appear at all. And that's why one of my suggestions, I was also once in the Kamm guideline, was a meeting with the patient representatives beforehand. I thought that was very, very good. And that the coordination processes of these patient representatives could also be well considered." |  |
|  | wish for incentives/financial support |  | wished financial support for participation (e.g. opinion on financial incentives) | "Otherwise I would of course, if you incur costs for travel and so on, it would of course be desirable if that were reimbursed. " |  |
|  | wish for appreciation |  | wished appreciation in advance or during the CPG development; feeling of being valued/appreciated for participation | "And of course, I would have really appreciated it if someone had asked, “How do you see it from your perspective?” If someone had asked that question so clearly, I would have found it very appreciative. But as I said, I'm not familiar with that kind of direct approach. More generally, yes, but not so directly." |  |
|  | other wishes |  | other wishes regarding PI in guidelines and guideline development | "In other words, improved documentation. I would like to see that. It would also be good if an abridged version of all guidelines were produced, also for laypeople and for a patient version. Guidelines are incredibly important and interesting, but completely uninteresting and incomprehensible for laypeople and for the actual users." |  |
