# Supplementary material for: Patient Involvement in the Development of Clinical Practice Guidelines – An Interview Study on the Experiences of Patients and Their Representatives in Germany
Source: Clin Public Health Guidel. 2026 Aug 10;3(4):e70081. doi: 10.1002/gin2.70081 (PMC13618489; doi:10.1002/gin2.70081)
Supplement: Supplementary file 4 [file GIN2-3-e70081-s003.docx]

**Supplementary Material 2 - Research team characteristics**

| **Characteristic** | **Description** |
| --- | --- |
| Credentials | All senior authors (KG, JB, DP) hold doctoral degrees. CO and SPH are currently doctoral candidates. |
| Occupation | CO, SPH and DP work as researchers at the Brandenburg Medical School. KG works as researcher at the Witten/Herdecke University. At the time of the interviews, JB worked as researcher at the Witten/Herdecke University. |
| Gender | CO, SPH, KG and JB are female. DP is male. |
| Experience and training | CO, SPH, KG, JB and DP are health services researchers. SPH, KG, JB and DP have co-authored multiple publications focusing on quantitative or qualitative research methods in health services research. CO has little experience in conducting qualitative interviews (therefore trained by an experienced colleague in advance and supported by experienced research team). |
| Relationship between participants and researchers | There was no connection between the interviewer and the participants. They did not know each other, and there were no further dependencies. Some recruited participants based on existing contacts with patient representatives within the research team (JB). |
| Participant knowledge of the interviewer | Participants did not know the interviewer. They knew she was a researcher at Brandenburg Medical School. |
| Interviewer characteristics | The main interest of CO in the topic was based on previous research on patient involvement in the development of clinical practice guidelines. |
